# Treatment for Overgrowth of a Finger Owing to Vascular Malformations: A Case Report

**DOI:** 10.7759/cureus.74716

**Published:** 2024-11-29

**Authors:** Sara Sugiura, Toru Sasaki, Koji Fujita, Toshitaka Yoshii

**Affiliations:** 1 Department of Orthopaedic and Spinal Surgery, Graduate School of Medical and Dental Sciences, Tokyo Medical and Dental University, Tokyo, JPN; 2 Department of Functional Joint Anatomy, Graduate School of Medical and Dental Sciences, Tokyo Medical and Dental University, Tokyo, JPN; 3 Division of Medical Design Innovations, Open Innovation Center, Institute of Research Innovation, Tokyo Medical and Dental University, Tokyo, JPN

**Keywords:** epiphysiodesis, finger overgrowth, proximal interphalangeal joint, timing of surgery, vascular malformation

## Abstract

Treatment for mild macrodactyly with only overgrowth of the solitary finger caused by vascular malformations (VMs) is rarely reported. We encountered a case of right middle finger overgrowth resulting from a VM in a seven-year-old girl. The length of her middle finger was 7.7 mm longer than her left middle finger. In order to control additional overgrowth, epiphysiodesis was planned when the finger reached the same length as that of her mother’s finger. At the age of 12, her right middle finger length reached the same length as her mother’s finger, and epiphysiodesis was performed. The length of her middle finger was 9.4 mm longer than her left middle finger. No additional overgrowth of the right finger occurred after surgery; however, the normal left finger stopped growing earlier than expected, maintaining a left-right difference of approximately 10 mm. No functional disorders were observed at a follow-up of 2.5 years. The timing of epiphysiodesis should be determined through a comprehensive assessment of multiple factors, not only the length of the parents’ fingers.

## Introduction

Limb overgrowth resulting from vascular malformations (VMs) is a symptom of certain syndromes, such as Proteus syndrome, Klippel-Trenaunay syndrome, and Parkes Weber syndrome, which are listed in the vascular anomaly classification by the International Society for the Study of Vascular Anomalies [1,2]. Proteus syndrome, Klippel-Trenaunay syndrome, and Parkes Weber syndrome are all infrequent diseases, and limb overgrowth due to VMs is an uncommon condition. A few cases of VMs on fingers have been reported, and some of these are associated with macrodactyly [3]. Recently, mutations in PIK3 have been reported to be found in macrodactyly, improving the importance of gene-targeted therapy [4]. On the other hand, orthopedic surgical supportive care is also important when functional impairment is a problem, and various approaches have been taken to address the diverse problems associated with macrodactyly. Due to its complexity, treatment usually requires a combination of multiple surgeries, and few reports have evaluated individual techniques [5]. This case report describes a mild macrodactyly associated with lymphatic malformation for which only an epiphyseal closure was performed.

## Case presentation

**Figure 1:**
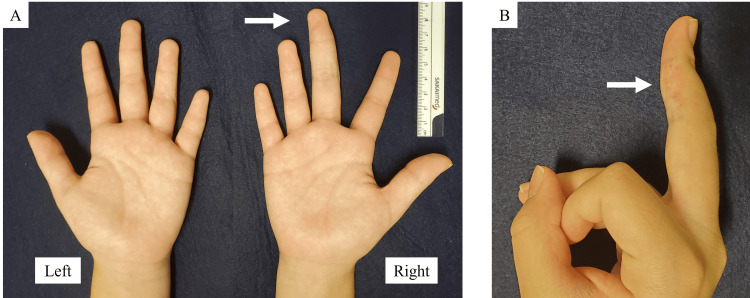
Macroscopic images of the patient’s finger at the first visit. (A) Compared with the other fingers, the patient’s right middle finger is long. (B) A hemorrhagic spot is observed on the finger.

**Figure 2:**
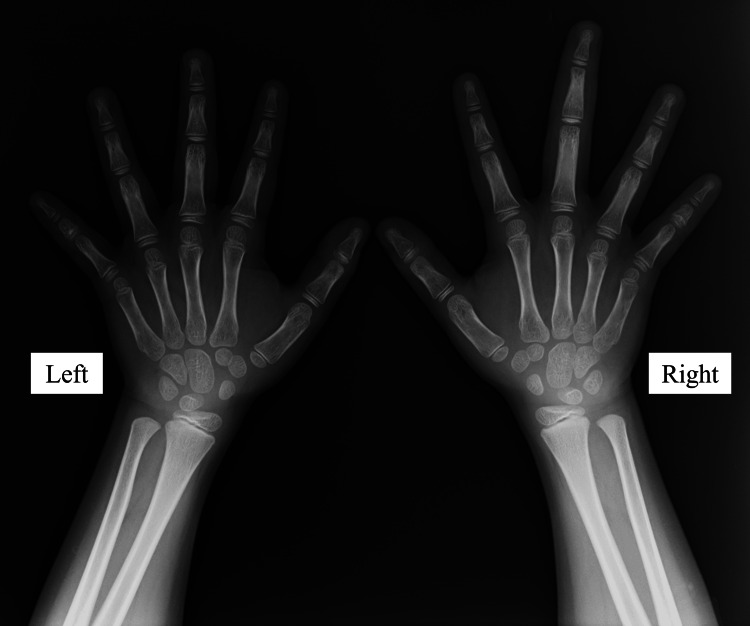
Radiographic images of the patient’s finger at the first visit. The phalanx of the middle finger is longer than that of the other fingers.

**Figure 3:**
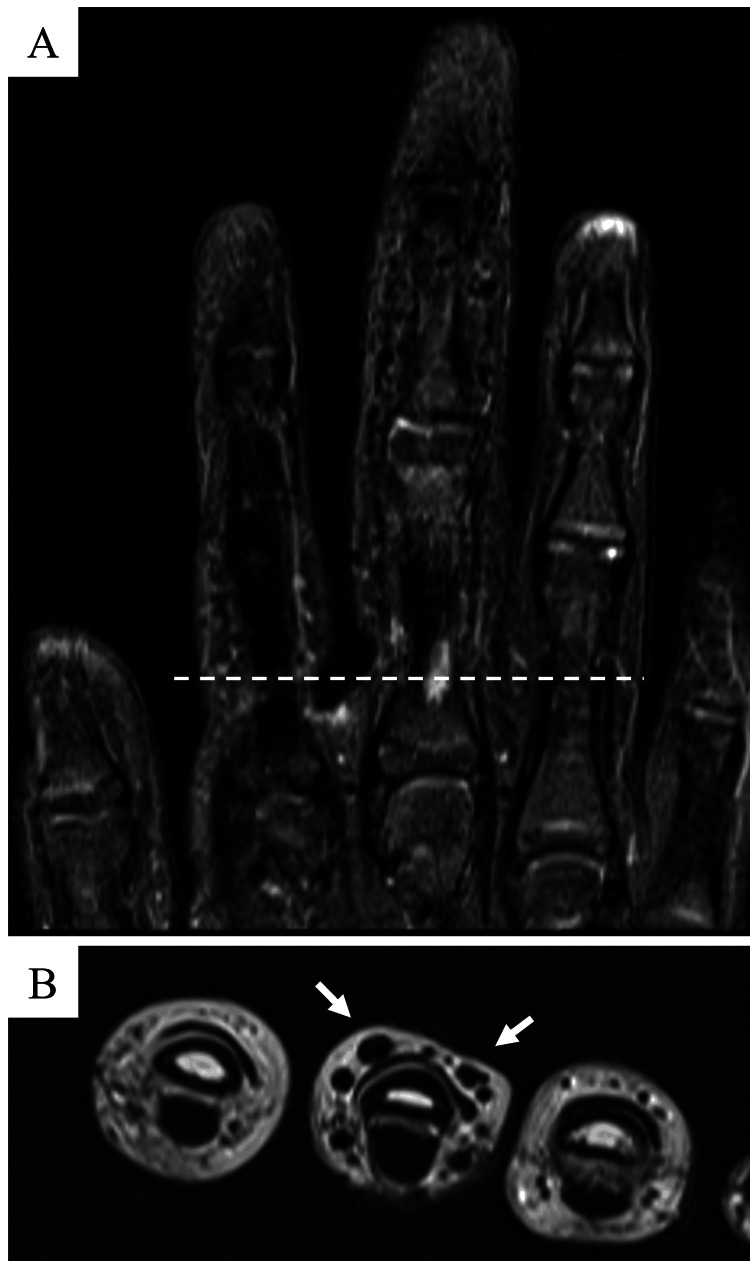
Magnetic resonance imaging of the patient’s finger. (A) Coronal image (fat-suppressed T2-weighted) of right fingers. (B) Axial image (T2-weighted) of right fingers. Magnetic resonance imaging revealed dilation of the arteries and veins, but no tumor was detected.

**Figure 4:**
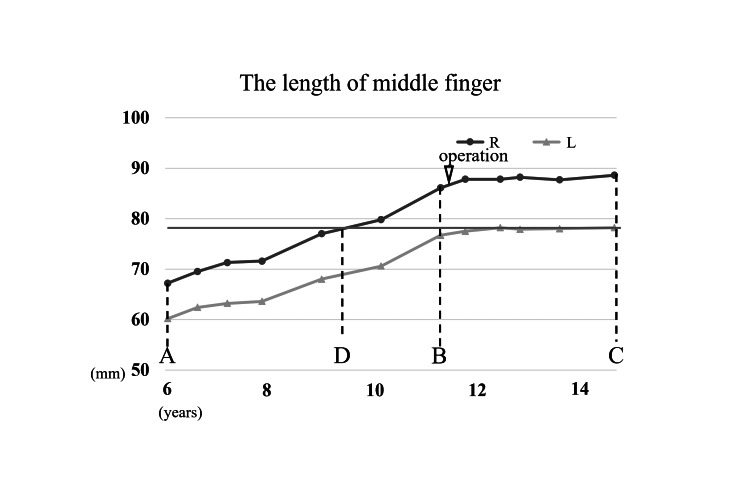
Graph of change in the length of the middle finger phalanx. (A)  At the first visit, the length difference is 7.7 mm (right: 67.9 mm; left: 60.2 mm). (B)  Before the surgery, the length difference is 9.4 mm (right: 86.1 mm; left: 76.7 mm). (C)  At the two-and-a-half-year follow-up, the length difference is 10.4 mm (right: 88.6 mm; left: 78.2 mm). (D) Approximately two years before the surgery, the length of the right finger reached the final length of the left finger (78.2 mm).

The patient was a seven-year-old girl who presented with a long right middle finger, a condition that she was aware of at the age of six years. In the same year, an internal hemorrhagic spot appeared distal to the proximal interphalangeal (PIP) joint of the right middle finger (Figure 1). The patient had repeated episodes of mild pain associated with internal bleeding, but this did not interfere with his daily life. She had no significant medical history, and her growth and development were normal. Her dominant hand was right. Her first menstruation was at age 11. Her mother’s height was 157 cm, and her father’s height was 168 cm, which was a few centimeters smaller than that of an average Japanese man. Blood tests, including coagulation abnormalities, were unremarkable. The length of her right middle finger phalanx, from the base of the proximal phalanx to the tip of the terminal phalanx by X-ray, was measured as 67.9 mm, while that of the left counterpart was measured as 60.2 mm (Figure 2). Ultrasonography revealed increased blood flow in the finger, whereas magnetic resonance imaging revealed vessel dilation without evidence of a tumor (Figure 3). The patient was diagnosed with overgrowth of the right middle finger attributed to a VM. The patient was followed up once a year with X-rays to determine finger length, and epiphyseal closure had not yet occurred. At the age of 12 years, when her right middle finger became the same length as that of her mother’s finger, the patient underwent epiphysiodesis. Her height at the time was 145 cm. The initial middle finger length difference was 7.7 mm (right 67.9 mm vs. left 60.2 mm), and the difference progressively increased to 9.4 mm (right 86.1 mm vs. left 76.7 mm) at surgery (Figure 4). The length of each phalanx (right/left) was as follows: 16.9/15.2 mm (distal), 24.4/21.1 mm (middle), and 40.4/36.4 mm (proximal). The transverse diameters of the left and right middle fingers at the largest point were 18.6 mm and 18.6 mm, respectively. There were no complaints of functional impairment.

**Figure 5:**
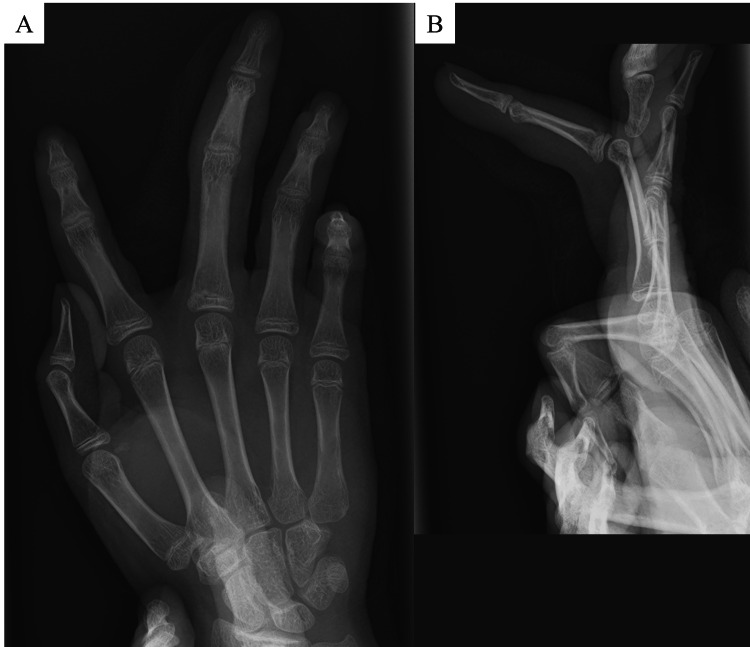
Postoperative X-ray of the right middle finger. (A) Postoperative X-ray (front view). (B) Postoperative X-ray (lateral view).

Epiphysiodesis was performed by removing the proximal and middle phalangeal growth plates using fluoroscopy. A skin incision was made on the dorsal sides of the metacarpophalangeal (MP) and PIP joints. The extensor hood was split to access the MP joint, and the transverse retinacular ligament was incised to expose the PIP joint. Using a 1.0-mm Kirshner wire and a 2.0-mm drill, a groove was created to excise the growth plate, followed by the application of an artificial bone graft with hydroxyapatite and atelocollagen to fill the cavity (Figure 5). No osteotomy was performed. Hemostasis of the subcutaneous vein was achieved through cauterization and compression. No histological test, including a gene-targeted test, was performed. In the first postoperative week, the patient’s forearm was immobilized to the phalanges using a splint to promote rest. Subsequently, range of motion exercises were performed.

After surgery, the right middle finger stopped growing, and no significant changes in VMs were observed. However, the growth of the patient’s healthy left middle finger was less than the expectation that the finger would grow as much as her mother’s finger. At the two-and-a-half-year follow-up after surgery, when she was 15 years old, her middle finger length was 78.2 mm (normal) in the left hand vs. 88.6 mm (elongated) in the right hand, and the gap of 10.4 mm remained (Figure 4). The transverse diameters of the left and right middle fingers at the largest point were 18.5 mm and 18.9 mm, respectively. Her height at the time was 152 cm. The grip strength was 20 kg in the left hand vs. 24 kg in the right hand, the range of motion of the PIP joint was 0-100 in the left hand vs. 0-100 in the right hand, and the range of motion of the DIP joint was 5 (hyperextension)-90 in the left hand vs. R 10 (hyperextension)-90 in the right hand. The Disability of the Arm, Shoulder, and Hand (DASH) score was 0, which meant no disability. The right middle finger remained elongated, but the patient complained of no inconvenience.

## Discussion

We encountered a case of middle finger overgrowth caused by a VM and performed epiphysiodesis, which resulted in good hand function. Based on episodes of internal bleeding and imaging findings, microcystic lymphatic malformation was the most probable differential diagnosis for VMs. Epiphysiodesis is a treatment for macrodactyly that is intended to control long-axis overgrowth. Ishida and Ikuta reported successful finger length control without additional surgeries in seven patients with macrodactyly who underwent epiphysiodesis at ages 0-13 and stated that treatment should be individualized [6]. Cerrato et al. reported the timing of epiphysiodesis for 17 fingers in seven patients with macrodactyly based on the length of the parent’s fingers [7]. Although it is difficult to evaluate the effect of epiphysiodesis on length control alone, because the surgery must be repeated for other problems such as increased phalangeal width and soft tissue hypertrophy, no major complications were reported in the above cases. Ezaki et al. also recommended awaiting surgery until the affected finger reached a length similar to that of the corresponding finger of the same-sex parent [5]. This case was a mild type of macrodactyly, with few factors other than increased length; therefore, we focused on length and performed the surgery on the basis of Ezaki et al.’s report in particular. In this case, we planned epiphysiodesis at a similar timing as in Ezaki et al.’s report, and there were no functional disorders and no additional surgery was necessary, but a 10 mm left-right length difference remained. In retrospect, the symmetry length could have been achieved if the surgery had been performed two years earlier. Determining the appropriate timing of surgery based solely on the finger length of the patient’s parent of the same sex may not always be optimal. In this case, the surgery should have been performed earlier, accounting for the below average height of the patient’s father. Since her father has a smaller-than-average height, she was likely smaller than her mother in adulthood. Her target height calculated from patients’ height was 156 cm, based on the report by Ogata et al. [8]. Her fingers would also likely be shorter than those of her mother; therefore, surgery could have been considered before the fingers of the healthy hand reached the same length as those of the mother’s hand. Various indicators, such as the parent’s height, individual’s menarche timing, epiphyseal line in other body parts, and individual’s growth curve, should be considered when determining the timing of surgery. If we aim to align the lengths of the left and right fingers, another possible option is an osteotomy at the time of surgery. In other cases, osteotomy may be indicated if the epiphyseal line closes during follow-up and there is a marked difference in finger length from right to left. However, this option is risky and may induce flexor and extensor tendon imbalances and associated finger deformities. Although no examination was performed in this case, some cases have mutations of PIK3CA, and treatment with PI3K inhibitors is a potential alternative [4]. For lymphatic malformation, sclerotherapy with an injection of OK-432 is a treatment option in Japan, but it is reported that sclerotherapy is often ineffective in microcystic lymphatic malformation [9]. In this case, no intervention was performed for the VM; only hemostasis was performed at the surgical site, and there were no complications, including additional overgrowth.

## Conclusions

This report presents a rare case of middle finger overgrowth caused by VM, which was considered microcystic lymphatic malformation. No additional overgrowth occurred after epiphysiodesis of the MP and PIP joints, and a left-right difference of approximately 10 mm remained. Determining the appropriate timing of surgery is crucial; in this case, the surgery should have been earlier, accounting for the height of the patient’s father being below average. Therefore, the timing of surgery should be determined through a comprehensive assessment of multiple factors related to the target height of patients, not only the lengths of the parents’ fingers.
